# Lectin Receptor-Like Kinases: The Sensor and Mediator at the Plant Cell Surface

**DOI:** 10.3389/fpls.2020.596301

**Published:** 2020-12-10

**Authors:** Yali Sun, Zhenzhen Qiao, Wellington Muchero, Jin-Gui Chen

**Affiliations:** Biosciences Division, Oak Ridge National Laboratory, Oak Ridge, TN, United States

**Keywords:** lectin, lectin receptor-like kinase, receptor-like kinase, plant defense, abiotic stress, biotic stress, plant development

## Abstract

Lectin receptor-like kinases (LecRLKs), a plant-specific receptor-like kinase (RLK) sub-family, have been recently found to play crucial roles in plant development and responses to abiotic and biotic stresses. In this review, we first describe the classification and structures of Lectin RLKs. Then we focus on the analysis of functions of LecRLKs in various biological processes and discuss the status of LecRLKs from the ligands they recognize, substrate they target, signaling pathways they are involved in, to the overall regulation of growth-defense tradeoffs. LecRLKs and the signaling components they interact with constitute recognition and protection systems at the plant cell surface contributing to the detection of environmental changes monitoring plant fitness.

## Introduction

Plants encounter various biotic and abiotic stresses during its growth and development. When plants are under biotic stress from bacteria, fungi, viruses, and herbivory insects, signatures on the surface of microbes, called pathogen-associated molecular patterns (PAMP), such as flagellin and elongation factor (EF-Tu), could be explicitly perceived by sets of receptors located at the plant cell surface, called pattern recognition receptors (PRRs) (Abdul Malik et al., 2020). The PAMP-signal perception of PRRs then initiates the first layer of plant innate immunity, called PAMP triggered immunity (PTI). PTI usually includes callose deposition, MAPK activation, calcium influx, reactive oxygen species (ROS) production, and salicylic acid accumulation (Jones and Dangl, 2006). A large number of receptor-like kinases (RLKs) and receptor-like proteins (RLPs) are deployed by plants as PRRs and often function as part of a multiprotein complex at the cell surface during PTI (He and Wu, 2016). One of the most well-studied plant PRRs is FLAGELLIN-SENSITIVE 2 (FLS2), an Arabidopsis RLK which recognizes the conserved 22 amino acids of bacterial flagellin (flg22) and forms a complex with its co-receptor BRASSINOSTEROID INSENSITIVE 1-associated receptor kinase 1 (BAK1) immediately after the perception to initiate PTI response (Macho and Zipfel, 2014). Generally, a plant RLK contains an extracellular domain for ligand perception, a transmembrane domain and an intracellular kinase domain, whereas an RLP lacks the cytoplasmic kinase domain and often functions together with RLKs (Ramonell et al., 2005; Wan et al., 2008). The extracellular domains of RLKs are highly variable, rendering their capabilities to perceive a great variety of signals. In addition to the recognition of PAMP signals, studies have shown that RLKs are involved in responses against abiotic stresses, plant-microbe symbiosis and the regulation of plant growth and development (Vaid et al., 2013; Sun et al., 2017; Tang et al., 2017). Depending on the structure of ectodomains, RLKs are divided into 17 classes (Wang J. et al., 2019). The Lectin RLK (LecRLK) class was named for its lectin/lectin-like ectodomain which can bind carbohydrates. While there is no homolog of LecRLKs found in the genomes of humans and fungi, LecRLKs are specifically widespread in plants (Vaid et al., 2013). There are 75 LecRLKs in Arabidopsis, 173 in rice, 231 in *Populus*, 198 in Eucalyptus, 189 in soybean, 38 LecRKs in *N. benthamiana*, 113 in potato, 46 in cucumber and 22 in tomato (Vaid et al., 2012; Wang et al., 2015; Yang et al., 2016; Liu et al., 2018; Lv et al., 2020; Zhang et al., 2020). Although the leucine-rich-repeat (LRR) RLK family is the largest and the most studied RLKs, recently LecRLKs have been emerging to be another key player of the RLK families, especially in plant innate immunity (Sun et al., 2017). Arabidopsis and rice are currently the two plant models where LecRLKs are well characterized. Although LecRLKs have been reported to be expressed in various plant tissues, including seed, root, stem, leaf and bud tissues, the specific function of many LecLRKs is still not well understood (De Hoff et al., 2009; Ezura et al., 2017). In this review, we analyze recent findings on the structure and function of LecRLKs and propose prospects of LecRLKs related research.

## Lectin Rlks Classification and Structures

**FIGURE 1:**
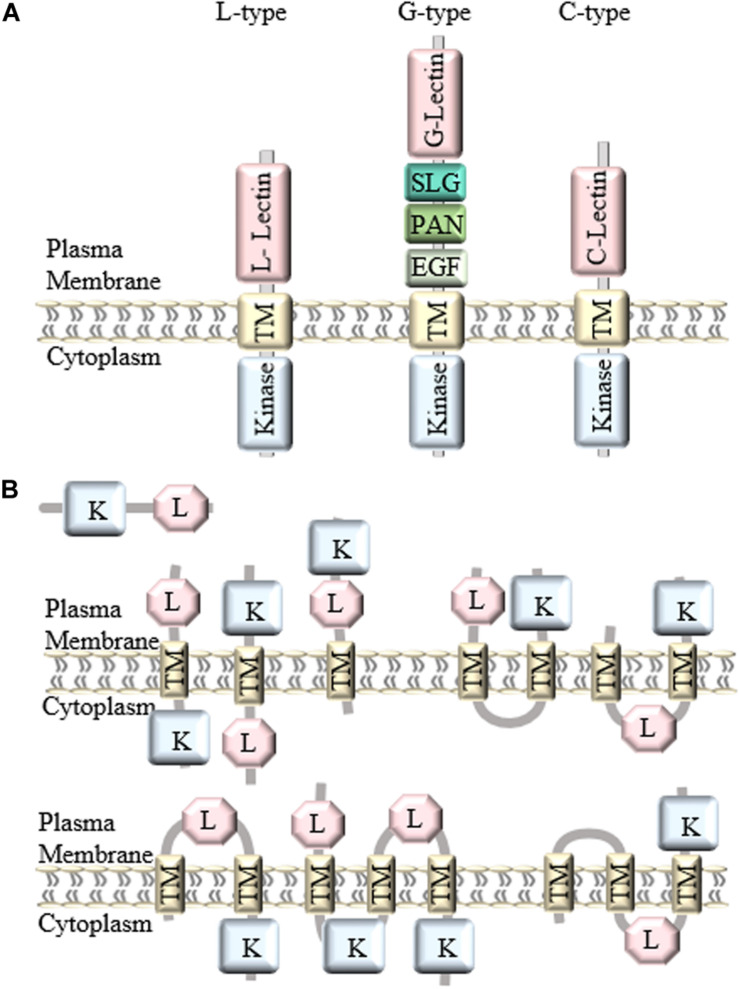
Classification and structure of LecRLKs. **(A)** There are three different types of LecRLKs based on the distinct extracellular domain: L-type LecRLKs with a legume like lectin domain, G-type LecRLKs with an α-mannose binding bulb lectin domain, an S-locus glycoprotein domain (SLG), a PAN and/or Epidermal Growth Factor (EGF) domain, and C-type LecRLKs with a calcium-dependent lectin domain. **(B)** Other predicted structures of LecRLKs. Some LecRLKs are predicted to have no transmembrane domain or kinase domain or have multiple transmembrane domains and different orientations.

In general, LecRLKs have three domains: an extracellular lectin domain, a transmembrane domain and a kinase domain. Based on the distinct extracellular lectin domain, they are classified into three types: L-type, G-type, and C-type (Figure 1A). There are 42 L-type, 32 G-type and 1 C-type LecRLKs in Arabidopsis, and 72 L-type, 100 G-type, and 1 C-type LecRLKs in rice, and 50 L-type, 180 G-type, and 1 C-type LecRLKs in *Populus* (Vaid et al., 2012; Yang et al., 2016).

### Lectin Domain

The first identified LecRLK is an L-type LecRLK in Arabidopsis (Herve et al., 1996). A typical L-type LecRLK contains a legume-like extracellular lectin domain (Wang and Bouwmeester, 2017). Although they share the typical β-sandwich fold structure, legume lectin proteins are soluble and present monosaccharide (glucose/fucose/mannose) binding specificity whereas L-type LecRLKs are plasma membrane-located and have a conserved hydrophobic cavity for binding to hydrophobic ligands, such as complex glycans, plant hormones and PAMPs (Bellande et al., 2017). In addition, legume lectin proteins are typically located in storage vacuoles, cytoplasm, the nucleus, and extracellular space, and are abundantly expressed in legume seeds (Van Damme et al., 2004). L-type LecRLKs contain the extended 17-aa residues formed loop at the C-terminal end of the lectin domain (Vaid et al., 2013). Moreover, during plant-microbe interactions, legume lectins can either bind to the carbohydrate moieties on the microbial cell wall or cell membrane and inhibit their growth at a distance away from the plant or favor the attachment of the bacteria to root epidermal cells during symbiosis (De Hoff et al., 2009). However, L-type LecRLKs can recognize extracellular signals and initiate complex signaling responses in the plant.

G-type LecRLKs contain an α-mannose binding bulb lectin domain, an S-locus glycoprotein domain (SLG), a Plasminogen/Apple/Nematode (PAN) domain and/or Epidermal Growth Factor (EGF) domain between the extracellular lectin domain and the transmembrane region (Tang et al., 2017). Compared to the structure of L-type LecRLKs, the G-type LecRLK lectin domain contains a β-barrel structure with 12 β-strands and shows potential binding affinity to α-D mannose, the detailed function of which is still not known (Vaid et al., 2013). S-locus domain is well-studied for its function in self-incompatibility (Kachroo et al., 2001). The PAN motif has been shown to participate in protein-protein interaction and protein-carbohydrate interaction, while the EGF domain has potential to be involved in disulfide bonds formation (Naithani et al., 2007).

C-type LecRLKs are the smallest group in plant LecRLKs with only one member found in rice, *Arabidopsis*, *Populus*, *Eucalyptus* and *Hydra vulgaris*, the function of which is still waiting to be elucidated. However, its calcium-dependent lectin domain has been found in mammalian proteins involved in innate immune response and self-/non-self-recognition (Reidling et al., 2000; Sanabria et al., 2010).

### Transmembrane Domain

Generally, LecRLKs have distinct lectin domains and contain more conserved transmembrane and cytoplasmic kinase domains. However, not all lectin kinases contain transmembrane domains. A genome-wide analysis of LecRLKs in *Populus* further divided PtLecRLKs into 8 classes, according to the prediction on transmembrane domains (TM) and the orientation of the other domains (Yang et al., 2016). For example, the first three classes all contain one TM domain, classes IV, V and VI all contain two TM domains, whereas classes VII and VIII contain three TM domains (Figure 1B). Although experimental studies need to be done to test the actual structure and orientation of LecRLKs, this study opened a new perspective of the structure and function of LecRLKs. The potential intracellular location of the lectin domain and extracellular location of the kinase domain prospect that LecRLKs might sense signals within the plant cell and trigger downstream signaling transduction in the apoplast, which may link to plant-environment interaction or the communication between plant cells. In addition, the TM domains of LecRLKs may also be involved in ligand recognition, which has been reported in other RLKs (Chen et al., 2006; Bi et al., 2016; Hohmann et al., 2017). Taken together, transmembrane domains of LecRLKs are not only required for the plasma membrane localization of LecRLKs, but may also be essential for the ligand recognition and signal transduction.

### Kinase Domain

Lectin receptor-like kinases were initially predicted to be Ser/Thr kinases because of the presence of DIKPAN and GT(FIL)GYIAPE within their kinase domain, and several studies have confirmed the Ser/Thr kinase activity of several LecRLKs (Herve et al., 1999). However, due to the findings that many other classes of RLKs show dual activities of both Ser/Thr kinase and Tyrosine kinase, there is still a possibility that some LecRLKs contain dual kinase activities (Taylor et al., 2016). Enzyme kinetic analysis showed that several divalent metal cations such as Mn^2+^ and Mg^2+^ could promote the autophosphorylation activity of LecRLKs and their general kinase activity (Nishiguchi et al., 2002; He et al., 2004). It points out that the kinase activity of LecRLKs might be related to the plant physiological homeostasis (He et al., 2004). Furthermore, the C-terminal tail of the kinase domain, containing a conserved xGxxx(V/I/L)P start and a GR doublet end, has been reported to be crucial for its catalytic activity and the interaction with downstream signaling molecules (Weiner and Zagzag, 2000). Collectively, kinase domain is crucial for the signal transduction of LecRLKs-mediated pathways. However, more enzymatic and biochemical analyses are required to clarify the activity of the kinase domain.

## LecRLKs in Plant-Microbe Pathogenic Interaction

**FIGURE 2:**
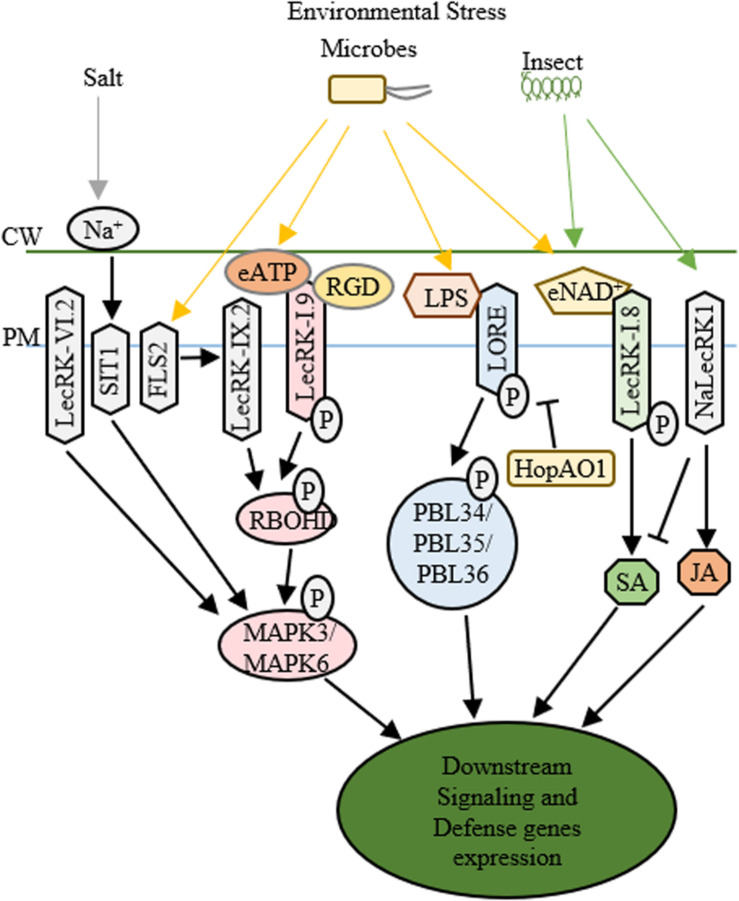
Functions of LecRLKs in plant stress responses. Environmental factors from abiotic and biotic stresses could be perceived by LecRLKs located at the plasma membrane, which can transduce the signal and trigger plant defense and stress responses. LecRK-IX.2 might serve as the downstream of FLS2. After it is activated, LecRK-IX.2 can phosphorylate RBOHD which then phosphorylates MAPK3/MAPK6 triggering defense genes expression. LecRK-I.9 could recognize external ATP (eATP) which then activates its autophosphorylation and kinase activity, causing the phosphorylation of RBOHD and MAPK3/MAPK6. The tripeptide Arg-Gly-Asp (RGD) ligand of IPI-O, a *Phytophthora infestans* effector, could be recognized by LecRK-I.9, which then disrupts its function in cell wall-plasma membrane adhesions and plant defense. SIT1 and LecRK-VI.2 can also activate MAPK3/MAPK6, while SIT1 is activated under salt stress, LecRK-VI.2 is involved in plant defense against several bacterial pathogens. After being activated by lipopolysaccharides (LPS), LORE undergoes autophosphorylation and phosphorylates cytoplasmic receptor kinases PBL34/PBL35/PBL36. *Pseudomonas syringae* effector HopAO1 could target LORE1 and suppress LORE1 triggered immune response. LecRK-I.8 perceives external NAD^+^ signal, triggers ROS burst and SA accumulation and induces defense genes’ expression. NaLecRK1 suppresses SA production and contributes to JA-mediated defense.

The characteristic lectin domain and kinase domain of LecRLKs provide a broad perspective on the cellular functions of LecRLKs (Bellande et al., 2017). The highly variable lectin domain implicates a wide range of ligands that LecRLKs may recognize. In addition, the phosphorylation activity of the kinase domain as the most common post-translational modification mechanism, projects a high number of downstream signaling LecRLKs can possibly transduce (Yin et al., 2019). As many other RLKs have been well characterized for their role in PAMP recognition and plant defense, increasing evidence support that LecRLKs are important players in the plant-microbe interactions (Figure 2).

The structural similarity to legume lectin proteins has implied the involvement of L-type LecRLKs in recognizing microbial surface signals. Several L-type LecRLKs have been reported to be activated by PAMP signal perception and trigger PTI and other plant responses against biotrophic pathogens. For example, an L-type LecRLK in Arabidopsis, LecRK-IX.2, serves as a positive regulator of PAMPs triggered immunity (Luo et al., 2017). First, pathogen infection of *Pseudomonas syringae* DC3000 activates the transcription of *LecRK-IX.2* in an FLS2-dependent pattern. Second, the overexpression plants of *LecRK-IX.2* exhibited a resistant phenotype to *P. syringae* DC3000 and enhanced ROS production and SA accumulation probably through the phosphorylation and activation of RbohD, a key player in PTI response. Finally, LecRK-IX.2 is required for the activation of flg22-induced PTI, indicating that LecRK-IX.2 likely acts downstream of FLS2 and upstream of SA accumulation. Whether LecRK-IX.2 directly interacts with FLS2 or its co-receptors such as BAK1/BIK1 and then gets phosphorylated by BAK1 or BIK1 still require further investigation.

Similar to LecRK-IX.2, LecRK-I.9, another L-type LecRLK in Arabidopsis known as DOES NOT RESPOND TO NUCLEOTIDES 1 (DORN1), also confers plant resistance to *P. syringae* DC3000, however, via downregulating MYC2-mediated JA pathway (Wang et al., 2016). The recent identification that DORN1 recognizes extracellular ATP (eATP) signals and directly phosphorylates RBOHD, triggering Ca^2+^ influx, MAPK activation, the accumulation of ROS, and defense gene expression has provided a thorough explanation of the function of DORN1 in plant defense (Choi et al., 2014; Chen et al., 2017; Wang L. et al., 2018). Additionally, LecRK-I.5, which shares 74% amino acid similarity to DORN1 but shows higher eATP binding affinity, has been identified to interact with DORN1 and synergically contribute to plant defense against bacterial phytopathogen *P. syringae* (Pham et al., 2020). These studies suggest that these L-type LecRLKs are involved in PTI, SA and JA signaling pathways and contribute to plant defense against biotrophic pathogens. Since PTI, SA and JA signaling cascades are internally correlated, manipulation of one of them might alter the other signaling responses. Because JA is well-known as a critical regulator in plant-necrotrophic pathogen interaction, it is not surprising to see that DORN1 participates in the plant defense against *Botrytis cinerea*, a necrotrophic pathogen (Tripathi et al., 2018).

Another example is LecRK-VI.2, an L-type LecRLK, which serves as a positive regulator of PTI and promotes plant defense against both biotrophic pathogen and necrotrophic pathogen such as *Pectobacterium carotovorum* and *Botrytis cinerea*. LecRK-VI.2 forms a complex with FLS2, specifically activates MAPK signaling cascade, induces the transcription of several PTI markers such a*s WRKY53 and FRK1*, and controls stomata closure during PTI (Singh et al., 2012; Wang L. et al., 2018). However, unlike LecRK-IX.2 or DORN1, LecRK-VI.2 shows no effects on early PAMP responses including ROS production, FLS2/BAK1 interaction or BIK phosphorylation in Arabidopsis, implying that LecRK-VI.2 works downstream of FLS2 and upstream of MAPK signaling (Singh et al., 2012, 2013). Ectopic expression of *LecRK-VI.2* in *N. benthamiana* confers resistance to both biotrophic and necrotrophic pathogens, but not fungi, and shows broad PTI responses such as ROS accumulation and callose deposition (Huang et al., 2014). These indicate that LecRK-VI.2 confers general plant defense against bacteria. Similarly, LecRK-V.2 and LecRK-VII.1 in Arabidopsis are involved in the JA-mediated stomatal immunity (Yekondi et al., 2018; Wang C. et al., 2019). Aside from L-type LecRLKs, G-type LecRLKs also participate in plant-microbe interactions. A G-type LecRLK in rice, OslecRK, has been reported to confer plant defense against BPH, blast disease and leaf blight disease (Cheng et al., 2013). *OslecRK* mutants are more susceptible to the infections of *Nilaparvata lugens*, *Magnaporthe grisea*, and *Xanthomonas oryzae pv. oryzae* compared to wild type, and mRNA levels of several defense-related genes such as *PR1, LOX2, and CHS* are decreased in *OslecRK* mutants, suggesting that OslecRK could activate multiple signaling responses in plant innate immunity. The finding that OslecRK directly interacts with an actin-depolymerizing factor (OsADF) via its kinase domain provided insights into a mode of action in which OsADF relays the signal from OslecRK and then triggers downstream signaling responses. Taken together, LecRLKs may be involved in multiple and complex signaling pathways of plant defense against both biotrophic and necrotrophic pathogens.

After the early stage PAMP infection, pathogens could release type III effectors into host cells, which can trigger either a much higher amplitude of immunity (ETI) or susceptibility (ETS) (Grant et al., 2006). While PTI and ETI share a lot in common, whether LecRLKs also play a role in ETI is an interesting topic for investigation. For example, under the infection of *Phytophthora infestans*, silencing of *NbLRK1* showed delayed hypersensitive response (HR) which is a typical characteristic of ETI (Kanzaki et al., 2008). In addition to the potential roles in ETI, LecRLKs may act as the targets of virulence effectors in ETS. Many virulence effectors have been shown to directly target PTI components to inhibit immune responses or target essential players in plant metabolism to trigger host susceptibility. For example, HopB1 could cleave phosphorylated BIK1 to impair PTI. Excitingly, a recent study reported the *P. syringae* effector HopAO1 targets LORE, a G-type LecRLK. In the early stage of *P. syringae* infection, the perception of the bacterial lipopolysaccharide (LPS) triggers LORE auto-phosphorylation. Then phosphorylated LORE phosphorylates receptor-like cytoplasmic kinases PBL34/PBL35/PBL36 and activates immune responses (Ranf et al., 2015). In the late stage of *P. syringae* infection, HopAO1, is secreted into the host cells. HopAO1 could interact with and dephosphorylate LORE, which causes reduced phosphorylation of PBL34/PBL35/PBL36, suppresses LORE-PBL34/PBL35/PBL36 activated immune response, and triggers host susceptibility (Shang-Guan et al., 2018; Kutschera et al., 2019; Luo et al., 2020). Moreover, a *Phytophthora infestans* effector IPI-O has been reported to target Arabidopsis DORN1 through the tripeptide Arg-Gly-Asp (RGD) and disrupt its function in the maintenance of cell wall-plasma membrane (CW-PM) adhesions and host defense (Bouwmeester et al., 2011). These findings indicate that LecRLKs may participate in ETI and could serve as potential targets of virulence effectors conferring host susceptibility (ETS).

Along with their roles in the mediation of plant-bacterial interaction, LecRLKs have been reported to be involved in different plant-fungal interactions. Similar to bacteria, fungal cell wall is the interface of plant-fungal interaction. It is mainly comprised of chitin, α- and β- linked glucans and glycoproteins, many of which have been found to be PAMPs and could be recognized by host membrane-bound receptors triggering host immune responses. For example, chitin could be recognized by LYK5 and CERK1, a lysin motif receptor kinase and a LysM receptor kinase in Arabidopsis. LYK5 and CERK1 could form a heterodimer and induce plant immune response through the activation of MAPK cascade (Cao et al., 2014; Erwig et al., 2017). The characteristic carbohydrate-binding lectin domains of LecRLKs are believed to play crucial roles in carbohydrate signal perception and have much more critical contributions in fungal cell wall recognition. For example, an L-type LecRLK in *Haynaldia villosa*, LecRK-V, has been shown to confer resistance to wheat powdery mildew through the association with ROS production and SA pathway (Wang Z. et al., 2018). The expression levels of SA signaling-related genes (*TaPR1* and *TaPR2*) and the expression levels of ROS generating genes (*TaNOX*, *TaCAT* and *TaGST*) in the transgenic *LecRK-V* plants are all induced after the infection of *Bgt*. These findings suggest the involvement of SA signaling and ROS pathway in LecRK-V mediated fungal pathogen resistance. Second, chitin treatment and *Bgt* treatment could both induce the transcriptional activation of *LecRK-V*. Furthermore, a G-type LecRLK from rice, Pi-d2, has been reported to contribute to plant defense against the fungal pathogen *Magnaporthe grisea* (Chen et al., 2006; Li et al., 2015). A single amino acid substitution at position 441 from Isoleucine (I) to Methionine (M) within its transmembrane domain could differentiate the resistant Pi-d2 allele from the susceptible ones. Although this I441M amino acid substitution does not change the plasma membrane localization of Pi-d2, protein structural prediction assays have identified significant structural differences between the resistant Pi-d2 allele (I441) and the susceptible allele (M441). The altered TM structure may not be able to relay ligand recognition information from extracellular space into intracellular kinase domain. These findings indicate the important role of TMs in the action of LecRLKs in plant-fungi interaction. A recent genome-wide associated mapping on *Populus trichocarpa-Sphaerulina musiva* system identified two LecRLKs associated with differential host responses to this fungal pathogen (Muchero et al., 2018). The first one is an L-type LecRLK which was highly expressed in the resistant *Populus* genotypes and was specifically induced in resistant *Populus* genotypes under *S. musiva* attack. The latter one is a G-type LecRLK and is generally highly expressed in susceptible *Populus* genotypes, and *S. musiva* infection could not trigger significant transcriptional level changes of this G-type LecRLK (Muchero et al., 2018). When the lectin domains of these two LecRLKs were purified and incubated with cell wall fractions of *S. musiva*, the G-type LecRLK showed higher binding affinity to the cell wall fractions than the L-type LecRLK regardless of KOH treatment. Furthermore, both the G-type and L-type LecRLKs showed significantly higher cell wall binding affinities after the *S. musiva* cell wall fractions were treated by KOH, suggesting that the ligand recognition was dependent on the alkaline extractable cell wall fractions. However, the difficulties in bulk expression and purification of these glycosylated proteins make it challenging to biochemically characterize the functions of these LecRLKs, in particular ligand identification. In addition, further phenotypic analyses are required to confirm and specify their functions in plant resistance/susceptibility including the use of overexpression and knockout plants of *LecRLKs*. Collectively, these studies enlightened our understanding on the role of LecRLKs in general plant-fungal interactions and indicated the potential application of LecRLKs on host resistance to pathogenic fungi.

Similar to the involvement in plant-microbe interactions, it has been shown from several different plant species that LecRLKs are involved in insect egg perception and the perception of insect feeding. An L-type LecRLK in Arabidopsis, *LecRK-I.8*, was found to be locally upregulated at the transcriptional level upon the *Pieris brassicae* oviposition and egg extract (EE) treatment, while in *lecRK-I.8* mutant plant, EE treatment caused significant reduction of ROS, SA production, *PR1* expression and cell death (Gouhier-Darimont et al., 2019). Interestingly, LecRK-I.8 has been shown to bind to extracellular NAD^+^ (Wang et al., 2017). However, whether NAD^+^ reception by LecRK-I.8 is the trigger of plant immune responses against insect egg and what is the downstream target of LecRK-I.8 still require further investigation. A G-type LecRLK in *Nicotiana attenuata*, NaLecRK1, was also shown to be involved in the perception of insect feeding. *NaLecRK1* could be transcriptionally induced under the attack of *Manduca sexta*, which promotes the suppression of SA production and contributes to the accumulation of JA-mediated defense response (Gilardoni et al., 2011). Furthermore, the transcriptional level of *NaLecRK1* is downregulated by COI1, a key player in JA signaling. Collectively, these studies indicate that LecRLKs participate in insect elicitors perception and trigger cell signaling responses to different insect attacks, and that the expression level of LecRLKs is under tight control by extracellular and intracellular signals.

## LecRLKs in Plant-Microbe Symbiosis

In addition to various biotic challenges, plants could establish beneficial relationships with different microbes. LecRLKs have been reported to be involved in the plant-microbe symbiotic relationship. A G-type LecRLK in *Medicago truncatula*, *MtLecRK1;1*, has been found to be transcriptionally downregulated upon the addition of *Sinorhizobium meliloti* or Nod factors, while overexpression of *MtLecRK1;1* in roots show more nodules formed compared to control, suggesting that MtLecRK1;1 is involved in the legume-rhizobia symbiosis (Navarro-Gochicoa et al., 2003; Bouwmeester and Govers, 2009; De Hoff et al., 2009). However, although MtLecRK1;1 has key conserved residues involved in monosaccharide binding and molecular modeling predicted its capability of binding with Nod factors, there is no increase in Nod factor binding in roots when *MtLecRK1;1* is overexpressed without its kinase domain, suggesting that the full protein might be required for its ligand binding activity (Navarro-Gochicoa et al., 2003). As many other RLKs and RLPs often function as a multi-protein complex and legume lectins have also been reported to form dimers, potential homodimerization of LecRLKs and heterodimerization of LecRLKs with other RLKs or co-receptors also deserve to be considered during the functional analysis of LecRLKs. For example, in Arabidopsis, LecRKIII.1 and LecRKIII.2 have been reported to form homodimers through their C-terminal kinase domains and then function in drought and salt stress responses. Whether the kinase domain is required for its Nod factor binding capability and whether MtLecRK1;1 can bind to Nod factors require further protein-protein interaction studies to clarify. Moreover, a G-type LecRLK in *Populus*, PtLecRLK1, has recently been identified as a key player in the *Populus-L. bicolor* symbiosis (Labbe et al., 2019). The transcriptomic study has shown the transcriptional induction of *PtLecRLK1* by *L. bicolor* and overexpression of *PtLecRLK1* in Arabidopsis, which is a non-host for *L. bicolor*, could initiate the symbiosis between *L. bicolor* and Arabidopsis (Labbe et al., 2019). Although further studies are required to disclose the fungi signal perception and downstream targets and signaling responses mediated by PtLecRLK1, these findings demonstrated the key status of LecRLKs in host-fungi compatibility and provided insights on the further application of beneficial ectomycorrhizal fungi to crops.

## LecRLKs in Plant Abiotic Stress

Aside from biotic stresses, plants often encounter various abiotic environmental challenges, such as salt, drought, heat and cold. Salt stress reduces crop productivity in many ways, two main effects of which are osmotic stress and ionic toxicity. Several reports have presented the involvement of LecRLKs in plant responses against salt stress (Figure 2). A G-type LecRLK in *Medicago sativa*, GsSRK, could maintain Na^+^/K^+^ balance under salt stress potentially through ROS scavenging and the regulation of osmotic homeostasis (Sun et al., 2013, 2018). A *Pisum sativum* LecRLK, PsLecRLK, promotes tissue compartmentalization of sodium and ROS scavenging activity providing the alleviation of the ionic and osmotic environment during salinity stress (Vaid et al., 2015; Passricha et al., 2019). In addition, a rice LecRLK, Salt Intolerance 2 (SIT2) was identified for its role in salinity stress tolerance potentially through its function in Na^+^ extrusion by manipulating SOS pathway (Passricha et al., 2020). Furthermore, Salt Intolerance 1 (SIT1), another rice L-type LecRLK, could be activated by NaCl and mediate salt sensitivity via the activation of MPK3/MPK6 leading to higher ethylene production and downstream ROS accumulation (Li et al., 2014; Zhao et al., 2019). Although this L-type LecRLK has been predicted to bind monosaccharide or polypeptide, the rapid activation of SIT1 by external NaCl treatment, the requirement of SIT1 in the activation of MPK3/MPK6 by NaCl, and the requirement of its kinase activity for salt sensitivity imply that it plays an essential role in the signaling sensing and transduction of external Na^+^, either through directly getting activated by Na^+^ or sensing the signal released by Na^+^ (Li et al., 2014; Passricha et al., 2020).

In addition to salt stress, LecRLKs have been found to play key roles in other abiotic stresses. Arabidopsis LecRK-V.5, DORN1, *Populus nigra* PnLPK and pepper CaLecRK-S.5 have been reported to be involved in wounding response. CaLecRK-S.5 potentially involves the activation of MAPK cascade and ROS burst (Woo et al., 2016, 2020). Genome-wide analysis of the LecRLK family in fox tail millet has identified 18 LecRLKs which may participate in drought and heat stress through transcriptomic level analysis under drought or heat stresses (Yu et al., 2018).

Despite that LecRLKs are involved in diverse abiotic stress responses, the underlying molecular mechanisms remain to be fully illustrated. It has been proposed that abiotic stress signals are likely to be recognized by cell surface receptors and then are transduced to downstream factors. The involvement of ROS burst and MAPK cascade in abiotic stress responses are same as biotic stress responses, suggesting overlap in LecRLKs mediated biotic and abiotic stress responses. Distinctly, ABA has been shown to be a key player in abiotic stress response, therefore, it would be interesting to test the role of LecRLKs in ABA signaling. Furthermore, although ABA is not directly involved in plant defense, high level of ABA could inhibit SA-mediated plant immunity through the suppression on MAPK activation and promote JA biosynthesis and JA-dependent gene expression in plant defense against some necrotrophic pathogens (Adie et al., 2007; Mine et al., 2017). On the other hand, the accumulation of SA could block the downstream ABA signaling responses (Moeder et al., 2010). Altogether, the engagement of LecRLKs in the complex phytohormone signaling pathways presents its crucial position in both biotic and abiotic stress responses.

## LecRLKs in Plant Development

Compared to plant-environmental interaction, the role of LecRLKs in plant growth and development has not been investigated as much in detail. In rice, OslecRK, which has been mentioned earlier providing broad-spectrum innate immune responses, also contributes to seed germination through its signal transduction from OsADF toward the regulation of α–amylase genes (Cheng et al., 2013). In Arabidopsis, seed germination has been found to involve the A4 subfamily of LecRLKs, LecRKA4.1, LecRKA4.2, LecRKA4.3, and LecRKA4.4, which redundantly and negatively regulate ABA inhibition of seed germination without interfering with GA signaling (Xin et al., 2009). However, in rice, GA and BR but not ABA or JA, have been found to be able to induce the expression of OsLSK1 and contribute to the improvement of grain yield. Key players in GA biosynthesis and signaling pathway, OsKO1, OsKO, GA20ox2 and OsGID2, are also transcriptionally induced in OsLSK1 transgenic plants (Zha et al., 2009; Zou et al., 2015). Two other LecRLKs, OsSIK2 in rice and GsSRK in soybean can both be induced by ABA treatment and contribute to plant architecture, while the former also delays dark-induced senescence in rice (Chen et al., 2013; Sun et al., 2018). Moreover, OsLecRK-S.7 in rice and LecRK-IV.2 in Arabidopsis are required for pollen development and the former is also responsible for male fertility (Wan et al., 2008; Peng et al., 2019). Although most of these findings on the involvement of LecRLKs in plant development still need further investigation to provide a clear picture, one clue is that plant hormones, especially ABA and GA, are in close association with the function of LecRLKs in plant growth and development.

## Discussion and Future Direction

As a cell surface receptor, the extracellular lectin domain of LecRLKs is believed to have the capability to perceive extracellular signals and transduce the signals to initiate cellular responses. While L-type LecRLKs have a conserved hydrophobic cavity responsible for binding hydrophobic ligands like complex glycans, plant hormones or PAMPs and G-type LecRLKs have a potential α-D mannose-binding affinity, there are only a few LecRLK’s ligands reported in plants. DORN1 can directly perceive extracellular ATPs in Arabidopsis and also directly interact with the tripeptide Arg-Gly-Asp (RGD) of effector IPI-O (Bouwmeester et al., 2011; Choi et al., 2014). LecRK-I.8 could directly bind to extracellular NAD^+^, and LORE can directly recognize bacterial lipopolysaccharide (LPS) (Ranf et al., 2015; Wang et al., 2017). PAMPs such as LPS are the typical characteristics of the microbial cell wall and have been well studied in the perception by other RLKs, while eATPs, and eNAD^+^, as signals released from microbial invasion, were very challenging to identify. For LecRLKs involved in plant-microbe interaction, methods such as lectin binding assays, where purified LecRLK proteins is used to screen against microbial cell wall fractions, could provide a broad understanding on its roles in microbial recognition specificity (Hatakeyama et al., 2012). Further analysis methods such as Gas chromatography-mass spectrometry (GC-MS) and Nuclear magnetic resonance (NMR) could be approached to identify the three-dimensional glycan structure. In addition, glycan microarray has been developed for high-throughput protein-glycan binding screening to build a thorough glycan ligands profile of LecRLKs (Geissner and Seeberger, 2016). Known synthesized glycans are printed onto a solid glass slide, and purified LecRLK protein can be incubated with the slide to screen for potential binding which can then be detected through direct or indirect fluorescence methods (Geissner et al., 2019). The involvement of LecRLKs in danger-associated molecular patterns (DAMP), egg-associated molecular patterns (EAMP), and abiotic stress signal perception indicates that the ligands probably are more related to the apoplast metabolic level changes triggered by environmental stresses. Therefore, metabolomic profile analyses of the plant apoplast space during the early stage of these environmental stresses could potentially provide a good understanding of the signals perceived by LecRLKs. Comparative mass spectroscopy-based metabolome analysis of plant apoplastic wash fluid before and after the above-mentioned stress conditions could identify novel metabolic changes related to plant stress responses (Green et al., 2020). Taken together the transcriptional and translational profiles of LecRLKs during the early stage of these environmental stresses, metabolite composition analysis of apoplastic wash fluid could prospect signal changes associated with LecRLKs and plant-environment interaction. However, one technical limitation is that the current apoplast wash fluid extraction method is very time-consuming, can only provide small quantities of apoplast wash fluid, and cannot completely eliminate cytoplasmic contamination (Gentzel et al., 2019). Definitive structural determination using NMR analysis will require a larger amount extraction method or new alternative methods to provide sufficient apoplast wash fluid.

**FIGURE 3:**
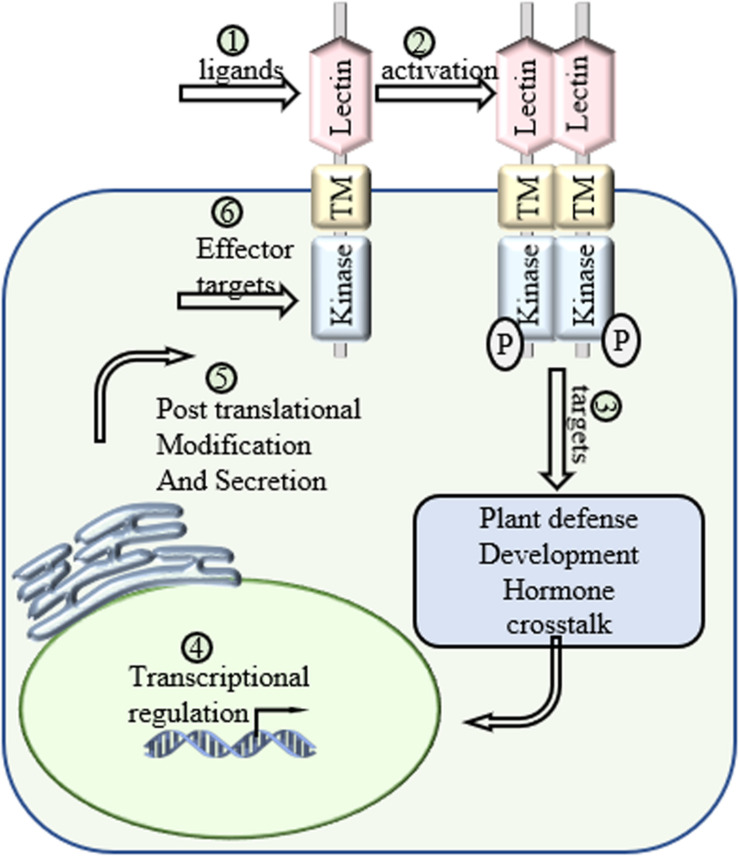
Future directions of LecRLKs. As a cell surface receptor and signal transduction mediator, LecRLKs can perceive extracellular signals and trigger intracellular responses to protect the plant. (1) Determination of the ligands of LecRLKs could clarify the recognition between plant-microbe and plant-environment at the molecular level. (2) The activation of LecRLKs after signal perception. Several findings indicate the formation of homodimer/heterodimer and auto-phosphorylation are required for LecRLKs’ kinase function. (3) The signaling pathways LecRLKs are involved in. Identifying their direct targets could be a very meaningful solution. (4) Transcriptional regulation of *LecRLKs*. Exploration of their dynamic transcriptional regulation mechanism could explain how they are induced during stress and help define their roles in plant growth and development. (5) LecRLKs have been found to be phosphorylated and N-glycosylated. Determination of post-translational modification could help our understanding of the functional dynamics of LectRLKs. (6) Some LecRLKs serve as key PRRs and are involved in PTI. There is a very high potential that virulence effectors might target them to suppress host immunity.

After the signal perception, the next question is the status of LecRLKs and how LecRLKs transduce the perceived signal. Many findings have shown the activation of LecRLKs after signal perception, but the activation mechanisms are still not well characterized (Figure 3). Proteomic profile analysis involves not only the identification of peptides or proteins, but also their post-translational modifications (Pang et al., 2020). Since most LecRLKs contain kinase activity, comparative phosphoproteomic profile analysis could locate and quantify phosphopeptides, then identify and predict proteins targeted by LecRLKs. Methods such as Immunoprecipitation-Mass Spectrometry (IP-MS) will be helpful to determine direct interaction targets when transfected protoplasts expressing tagged LecRLKs are available. Then protein-protein interaction detecting methods, such as yeast 2-hybrid, bimolecular fluorescence complementation (BiFC) and co-immunoprecipitation (co-IP), can be used to verify their association and interaction, and enzymatic activity tests can be adopted to verify the candidate’s role as the phosphorylation substrate and identify the enzymatic specificity of LecRLKs. This is a fruitful area that is worth of further investigation. LecRLKs have been shown to be involved in hormone signaling pathways such as SA, JA, ABA, GA, Auxin, and ET, either during plant development or during plant stress responses. First, several LecRLKs could affect the accumulation of phytohormones through regulating key phytohormone synthesis genes or other key players in the signaling pathway. Phytohormones could also affect the level of LecRLKs through transcriptional reprogramming. Transcriptomic profile analysis could provide a broad understanding in the expression level of key players in phytohormone signaling associated with LecRLKs. Second, since L-type LecRLKs have a conserved hydrophobic cavity and several plant hormones, such as ET and Auxin, have been shown to bind to the hydrophobic pocket of its receptors, definitive structural analysis such as HPLC-mass spectrometry, NMR and X-ray crystallography can help define whether LecRLKs could recognize plant hormones. Third, with phytohormones as the linker between LecRLKs and downstream signaling responses, these studies can lead to more explorations on the role of LecRLKs in plant development and plant stress responses. Most of plant development and cell signaling processes consume energy. Plants use their roots and leaves to collect chemical energy and nutrition through direct absorption and photosynthesis from the environment, which then is distributed to metabolic processes for growth/development or defense. When plants are under stresses, complex hormone crosstalk has been reported to fine-tune the growth-defense tradeoff (Huot et al., 2014). LecRLKs have also been shown to be a crucial player in both plant growth/development and plant defense. During the naïve status, LecRLKs regulate plant growth/development such as root and pollen development. When under environmental stresses, LecRLKs sense those extrinsic signals and initiate specific responses. Typically, plants encounter multiple environmental challenges simultaneously. When these different biotic and abiotic signals are simultaneously perceived by LecRLKs, the question is how plants integrate them and then process the priority of growth and defense to maximize plant fitness (Figure 3). For example, pathogen attack causes the reduction of extracellular ATPs which then triggers moderate defense responses, while eATP is also involved in alleviating the decrease of photosynthesis under pathogen infection (Chivasa et al., 2009; Sun et al., 2012; Choi et al., 2014; Tatagiba et al., 2016). As the only eATP receptor found so far, DORN1 has a high probability to play a role in this growth-defense tradeoff in plants.

Following the specific cellular responses triggered by the activation of LecRLKs, the complex signaling responses, in turn, affect the expression of LecRLKs. The basal expression levels of LecRLKs vary across tissues and organs, as well as developmental stages. LecRLKs could be highly induced by different environmental stimuli or during specific developmental stages. Defining the dynamic transcriptional regulation mechanisms of LecRLKs will be very helpful for providing a broader view of the roles of LecRLKs. Comprehensive transcriptomic profile analysis on the level of LecRLKs during different stages of environmental stresses could point out the role of LecRLKs, identify its co-expressed associates dynamically and depict a broader and consecutive perspective of stress responses but not just a snapshot of the process. Moreover, recent proteomic data analysis predicted the post-translational modification (PTM) of several Arabidopsis and *Populus* LecRLKs via phosphorylation and N-glycosylation (Yang et al., 2016; Bellande et al., 2017). More experimental data are emerging to support the PTM of LecRLKs. For example, Arabidopsis LecRK-IX.1 and LecRK-IX.2 have both been reported to be N-glycosylated (Wang et al., 2015). Since many PTMs have been shown to be responsible for protein trafficking, prediction and verification of the PTM of LecRLKs not only provides insights on the biochemical characterization of LecRLKs, but also clarifies our understanding on the intracellular trafficking and localization of LecRLKs. Taken together, the dynamic transcriptional, translational and post-translational level analyses will help decipher the overall regulations and modifications on LecRLKs.

## Conclusion

LecRLKs could sense self- and non-self-signals, mediate the signal transduction to contribute to plant growth/development such as root and pollen development, seed germination, grain yield and senescence, as well as plant defense against various environmental challenges from abiotic stresses of salt and drought to biotic stresses of bacteria, fungi and herbivory insects. In order to clarify the mechanisms LecRLKs utilize to regulate those stress responses, it is needed to decipher the signals LecRLKs perceive, the downstream players LecRLKs target to transduce the signal, and the signaling pathways LecRLKs activate or inhibit. While recent findings have begun to decode these processes, further investigations are required to present a clear perspective on the function and action of LecRLKs. Meanwhile, it has been found that the transcription of several LecRLKs is also under positive feedback control. The revelation of the dynamic transcriptional regulation and potential post-translational modification mechanism of LecRLKs could unravel their role in maintaining the plant physiological homoeostasis under the naïve state and environmental challenges. The knowledge presented by above-mentioned and future studies could favor plant breeding and engineering strategies in selecting the genetic traits to maximize plant fitness and crop yield under various environmental conditions.

## Author Contributions

YS drafted the manuscript. ZQ, WM, and J-GC revised the manuscript. All authors contributed to the article and approved the submitted version.

## Conflict of Interest

The authors declare that the research was conducted in the absence of any commercial or financial relationships that could be construed as a potential conflict of interest.
